# Transcutaneous auricular vagus nerve stimulation with task-oriented training improves upper extremity function in patients with subacute stroke: a randomized clinical trial

**DOI:** 10.3389/fnins.2024.1346634

**Published:** 2024-03-08

**Authors:** Meng-Huan Wang, Yi-Xiu Wang, Min Xie, Li-Yan Chen, Meng-Fei He, Feng Lin, Zhong-Li Jiang

**Affiliations:** ^1^School of Rehabilitation Medicine, Nanjing Medical University, Nanjing, Jiangsu, China; ^2^Department of Rehabilitation Medicine, The First Affiliated Hospital of Nanjing Medical University, Nanjing, Jiangsu, China; ^3^Department of Rehabilitation Medicine, Sir Run Run Hospital, Nanjing Medical University, Nanjing, Jiangsu, China

**Keywords:** transcutaneous auricular vagus nerve stimulation, task-oriented training, motor evoked potentials, functional near-infrared spectroscopy, stroke, upper extremity rehabilitation

## Abstract

**Background:**

Transcutaneous auricular vagus nerve stimulation (taVNS) has emerged as a promising brain stimulation modality in poststroke upper extremity rehabilitation. Although several studies have examined the safety and reliability of taVNS, the mechanisms underlying motor recovery in stroke patients remain unclear.

**Objectives:**

This study aimed to investigate the effects of taVNS paired with task-oriented training (TOT) on upper extremity function in patients with subacute stroke and explore the potential underlying mechanisms.

**Methods:**

In this double-blinded, randomized, controlled pilot trial, 40 patients with subacute stroke were randomly assigned to two groups: the VNS group (VG), receiving taVNS during TOT, and the Sham group (SG), receiving sham taVNS during TOT. The intervention was delivered 5 days per week for 4 weeks. Upper extremity function was measured using the Fugl-Meyer Assessment-Upper Extremity (FMA-UE), the Action Research Arm Test (ARAT). Activities of daily living were measured by the modified Barthel Index (MBI). Motor-evoked potentials (MEPs) were measured to evaluate cortical excitability. Assessments were administered at baseline and post-intervention. Additionally, the immediate effect of taVNS was detected using functional near-infrared spectroscopy (fNIRS) and heart rate variability (HRV) before intervention.

**Results:**

The VG showed significant improvements in upper extremity function (FMA-UE, ARAT) and activities of daily living (MBI) compared to the SG at post-intervention. Furthermore, the VG demonstrated a higher rate of elicited ipsilesional MEPs and a shorter latency of MEPs in the contralesional M1. In the VG, improvements in FMA-UE were significantly associated with reduced latency of contralesional MEPs. Additionally, fNIRS revealed increased activation in the contralesional prefrontal cortex and ipsilesional sensorimotor cortex in the VG in contrast to the SG. However, no significant between-group differences were found in HRV.

**Conclusion:**

The combination of taVNS with TOT effectively improves upper extremity function in patients with subacute stroke, potentially through modulating the bilateral cortex excitability to facilitate task-specific functional recovery.

## 1 Introduction

A cerebrovascular accident (CVA), often referred to as a stroke, arises from an interruption of blood flow or bleeding in a region of the brain, resulting in impaired brain function. According to the Global Burden of Disease (GBD) study, stroke is the primary cause of mortality among Chinese adults and the second leading cause of death worldwide (Zhou et al., 2019; GBD 2019 Stroke Collaborators, 2021). Reports indicate that 55–75% of stroke patients continue to experience upper extremity motor dysfunction within 3–6 months of onset (Kwakkel et al., 2003). Upper extremity motor dysfunction markedly influences the prognosis of patients, impacting their mobility, daily activities, and overall quality of life (Lai et al., 2002; Nichols-Larsen et al., 2005). For poststroke upper extremity rehabilitation, the task-oriented training (TOT) involves structured movement training based on daily activities to engage patients actively, thereby promoting motor function during the targeted task practice (Yoo and Park, 2015). Motor priming, a type of implicit learning wherein external stimulation prompts changes in the motor cortex and behavior, has been reported in motor skill learning recently (Jin et al., 2019). Previous research demonstrated that stimulation-based priming combined with TOT could facilitate brain reorganization and enhance upper extremity dexterity (Higgins et al., 2013; Alsubiheen et al., 2022). The most commonly used non-invasive brain stimulation techniques include transcranial magnetic stimulation (TMS) and transcranial direct current stimulation (tDCS) (Chhatbar et al., 2017; Bai et al., 2022; Zhang et al., 2022). However, the application of TMS and tDCS is limited by the need for high precision requirements and complex operations.

Vagus nerve stimulation (VNS) acts as a promising brain stimulation-based priming technique involving various forms of stimulation applied to the vagus nerve network. By regulating the balance of the autonomic nervous system and targeting neuroprotective and neuroplasticity pathways, VNS holds potential as a therapeutic tool in various neurological and psychiatric conditions (Ma et al., 2019). A multicenter, randomized, double-blind trial (VNS-REHAB) conducted by Dawson et al. (2021) confirmed the effectiveness of implanted VNS (iVNS) paired with upper extremity training in patients with ischemic stroke. However, the application process of iVNS may carry a high rate of potential complications due to its invasiveness (Ma et al., 2019). Recently, transcutaneous auricular VNS (taVNS), as a safe and easy-to-use neuromodulation technique, has been proposed, which utilizes transcutaneous stimulation of the cymba conchae and tragus innervated by fibers of the auricular branch of the vagus nerve (ABVN). Neuroimaging studies have demonstrated that taVNS can elicit brain activation effects similar to iVNS (Badran et al., 2018). Several studies (Capone et al., 2017; Redgrave et al., 2018; Wu et al., 2020; Chang et al., 2021; Li et al., 2022) have combined taVNS with regular rehabilitation training or robot-assisted arm training to improve upper extremity function in hemiplegic patients. To ensure consistency in taVNS research, an international consensus has been established for minimum reporting standards, which reported that a signal with a pulse width between 200 and 300 μs at 25 Hz, and a duty cycle of 30 s on, 30 s off has often been adopted in studies (Farmer et al., 2021). Nonetheless, the mechanism by which taVNS achieves task-specific benefits in upper extremity function remains unclear. In this study, we hypothesized that combining taVNS with TOT simultaneously would present a novel approach to enhance upper extremity function recovery in stroke patients. To assess the immediate and long-term effects of taVNS on hemodynamics and cortical excitability, we employed functional near-infrared spectroscopy (fNIRS) and single-pulse transcranial magnetic stimulation (TMS), respectively. The primary objective of this study was to investigate the effects and potential mechanisms of taVNS paired with TOT in facilitating the recovery of upper extremity function in patients with subacute stroke.

## 2 Materials and methods

### 2.1 Participants

The stroke patients with upper extremity motor dysfunction were recruited from the Department of Rehabilitation Medicine at Sir Run Run Hospital of Nanjing Medical University between February 2020 and December 2022. The inclusion criteria were as follows: (1) first-time occurrence of a unilateral supratentorial stroke confirmed by computed tomography (CT) or magnetic resonance imaging (MRI) within 6 months of onset; (2) presence of unilateral hemiplegia of the upper extremity; (3) upper extremity impairment ≥ third level in the Functional Test for the Hemiparetic Upper Extremity (FTHUE), and FMA-UE scores ranged from 20 ~ 50; and (4) normal cognitive function with the ability to follow instructions and complete the study. The exclusion criteria were as follows: (1) presence of implanted electronic devices, intracerebral vascular clips, or other electrically activated or sensitive support systems; (2) presence of abnormal skin conditions that may interfere with the stimulation or the stimulation device, such as scar tissue, broken skin; (3) presence of severe cardiovascular, pulmonary, or advanced diseases affecting other systems; (4) previous impairment of the vagus nerve; (5) upper extremity dysfunction caused by reasons other than stroke; (6) use of neuropsychotropic drugs such as antidepressants or benzodiazepines; (7) Botox injection within the past 3 months; (8) resting heart rate below 60 beats per minute; and (9) presence of asthma, tumors, or severe dysphagia. The trial was performed in accordance with the principles of the Declaration of Helsinki and received approval from the Ethics Committee of Sir Run Run Hospital, Nanjing Medical University (No. 2020-SR-001). All participants signed informed consent. Further details regarding the subjects can be found in Table 1.

**Table 1:** Demographics and baseline clinical characteristic.

Characteristics	Groups		
	VNS group (*n* = 20)	Sham group (*n* = 20)	*p*-value
Age (years)	55 (11)	57 (11)	0.515^a^
Gender			
male	18 (90)	15 (75)	0.407^b^
female	2 (10)	5 (25)	
Stroke onset (months)	3.20 (2.04)	4.15 (1.60)	0.074^a^
Hemiparetic side			
left	9 (45)	8 (40)	0.749^c^
right	11 (55)	12 (60)	
Stroke type			
ischemic	13 (65)	14 (70)	0.736^c^
hemorrhagic	7 (35)	6 (30)	
FMA-UE	31 (9)	30 (9)	0.665^d^
ARAT	16 (11)	15 (11)	0.968^d^
FTHUE	5.85 (2.06)	6.05 (2.76)	0.945^a^
MBI	72 (17)	69 (15)	0.543^a^
HR	83 (11)	79 (12)	0.271^a^

^a^Welch two sample t-test; ^b^Fisher’s exact test; ^c^Pearson’s Chi-squared test; ^d^Wilcoxon rank sum test. FMA-UE,Fugl-Meyer assessment-upper extremity; ARAT, Action Research Arm Test; FTHUE, Functional Test for the Hemiparetic Upper Extremity; MBI, Modified Barthel Index; HR, average heart rate.

### 2.2 Study design

**Figure 1:**
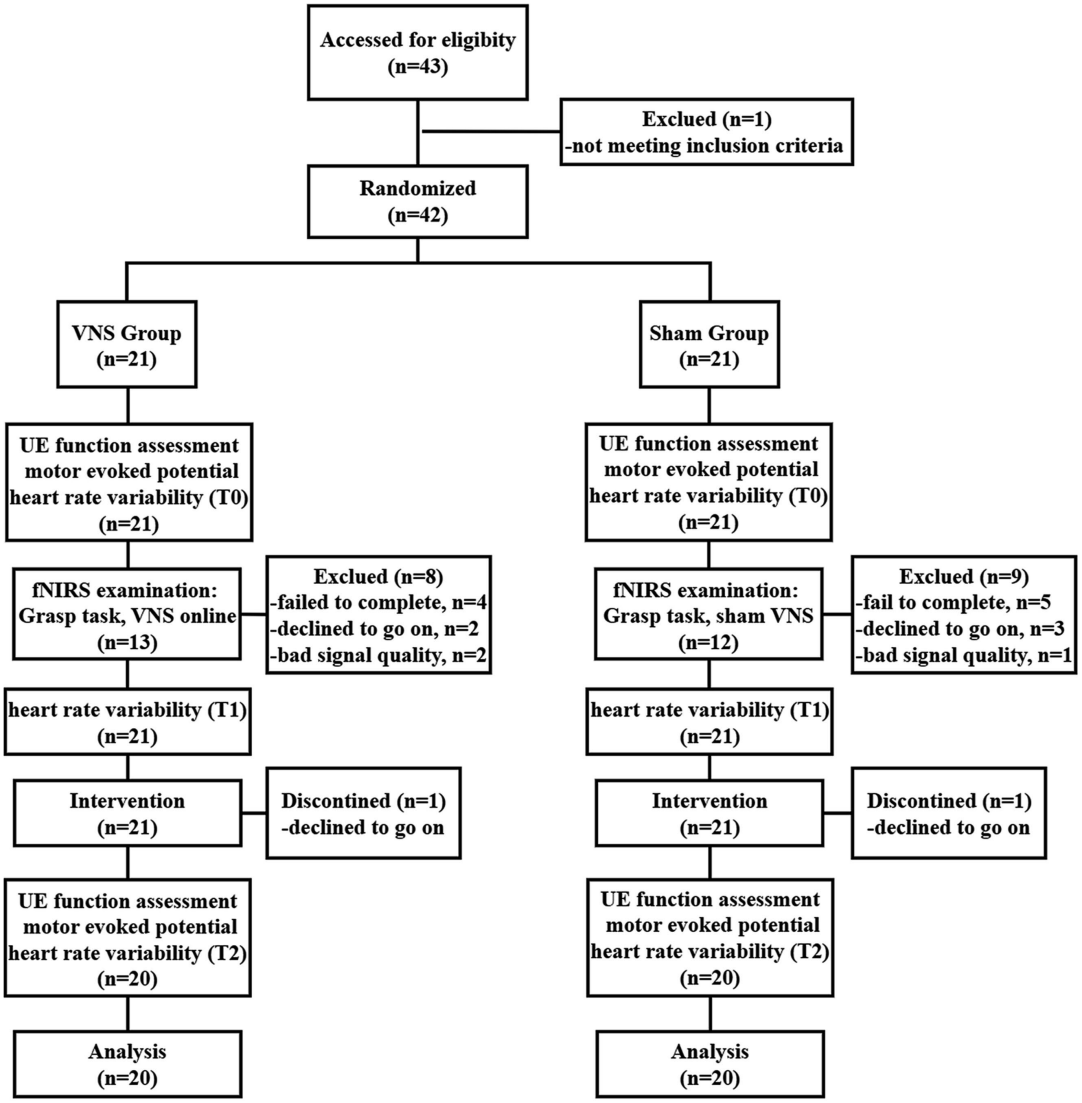
Study flow diagram. VNS: vagus nerve stimulation; UE, Upper extremity; fNIRS, Functional near-infrared spectroscopy.

This pilot study was designed as a randomized, double-blind, sham-controlled clinical trial, which followed the CONSORT checklist (Supplementary material S1). The patients were randomly assigned (1:1) to either the VNS group (VG) or the Sham group (SG) using a random number table. The patients in the VG received real taVNS during 1 h of task-oriented training, while the patients in the SG received sham taVNS during task-oriented training. The treatment was delivered 5 ays per week for 4 weeks. To minimize subjective bias, different individuals performed the roles of the TOT therapist, taVNS operator, and outcome assessor. To explore the immediate effect of taVNS, an fNIRS examination with HRV assessment was conducted before the first treatment session. The study flow is shown in Figure 1.

### 2.3 Intervention

#### 2.3.1 Transcutaneous auricular vagus nerve stimulation (taVNS)

The taVNS treatment was delivered simultaneously with TOT for 1 h in accordance with Zhang et al. (2023), using the Auricular Vagus Nerve Stimulator (tVNS501, RISHENA Co., Ltd., Changzhou, China). A specialized earphone with two dot-like electrodes delivered electric stimulation to the left auricular cymba concha of patients in the VG. Conversely, patients in the SG wore the same earphones without electrodes. Although they could observe the current reading on the stimulator, no actual current was delivered, resulting in ineffective stimulation. Before stimulation, the left auricular concha of each patient was cleaned using alcohol wipes. According to the international consensus for minimum reporting standards (Farmer et al., 2021), the stimulation parameters were configured as follows: 500 μs square pulses at 25 Hz for 30 s, with a duty cycle of 1:1. The current intensity was individually adjusted to a tolerable level for each patient, ensuring that it did not exceed 10 mA (with an average of 6.55 ± 1.57 mA in the VG) (Capone et al., 2017; Redgrave et al., 2018; Wu et al., 2020; Li et al., 2022).

#### 2.3.2 Task-oriented training (TOT)

Both groups of patients underwent TOT practice, which was supervised or assisted by a well-trained occupational therapist. The TOT practice involved eight exercises, requiring patients to perform as many trials as possible in each session. Each session of task-oriented training lasted for 1 h. The occupational therapist was allowed to adjust the intensity of each exercise based on the patient’s training objectives and functional performance. The detailed steps of the TOT were as follows (Kim et al., 2013; Tang et al., 2014): (1) instruct patients to place their hand on a table with adjustable height; (2) instruct patients to touch the tip of their nose; (3) instruct patients to rotate their forearm and alternate the palm upwards and downwards; (4) instruct patients to bend the elbow to a 90-degree angle, position the forearm neutrally, and extend the wrist joint to touch a designated object on the table as far as possible; (5) while maintaining the same position as in step (4), instruct patients to grip and release a water bottle placed on the table; (6) instruct patients to pick up a cup from the table, simulating a drinking motion, then return it to its original position; (7) instruct patients to pour peanuts from a cup onto a plate on the table without using compensatory trunk movements; (8) instruct patients to manipulate objects with the assistant of a hand-function robot (Gloreha Professional 2, Idrogenet, Italy).

### 2.4 Outcome measurements

The long-term outcomes were assessed using clinical scales and motor-evoked potentials (MEPs) elicited by single-pulse TMS. The Fugl-Meyer Assessment-Upper Extremity (FMA-UE) and the Action Research Arm Test (ARAT) were employed to evaluate the function of the hemiplegic upper extremity, and the modified Barthel Index (MBI) was used to evaluate the activity of daily living (Chen et al., 2021). The MEPs were applied to evaluate the cortico-spinal excitability. The primary outcome measure was the change score of the Upper Extremity Fugl-Meyer Assessment (UE-FMA). All outcomes were evaluated before the first treatment and after the final treatment. Additionally, in order to explore the feasibility of the current study and assess the immediate effect of taVNS during grasp tasks with the hemiplegic hand, an fNIRS examination was conducted before the first treatment session. HRV data were collected before and after the fNIRS examination (T0, T1) and after the last treatment (T2) to assess the effectiveness of taVNS on the vagus nerve network.

#### 2.4.1 Upper extremity function assessment

The Fugl-Meyer Assessment-Upper Extremity (FMA-UE) is a widely used tool for evaluating upper extremity impairment and coordination/speed in stroke patients. The FMA-UE comprises 33 items, each scored on a scale ranging from 0 to 2, culminating in a total score of 66 points (Gladstone et al., 2002; Rech et al., 2020).

The Action Research Arm Test (ARAT) is a standardized observational scale extensively used to assess the functional abilities of the upper extremity in stroke survivors, closely reflecting their daily activities. The ARAT consists of 19 items categorized into four subtests: grasping, gripping, pinching, and gross movement. Each item is scored on a scale from 0 to 3, with a maximum possible score of 57 (Hsieh et al., 1998; Zhao et al., 2019a, b).

The Modified Barthel Index (MBI) is a frequently used outcome measure to evaluate performance in activities of daily living (ADL) among stroke patients. The MBI comprises 10 items, with a total score of 100 (Shah et al., 1989).

#### 2.4.2 Motor-evoked potentials examination

Motor-evoked potentials (MEPs) refer to the action potentials elicited by single-pulse TMS of the primary motor cortex (M1), providing insight into cortico-spinal excitability. MEP latency refers to the time taken for the motor response to occur after the stimulation of the motor cortex. It is calculated by measuring the time interval between the onset of stimulation and the onset of the MEP waveform (Rossi et al., 2021; Vucic et al., 2023). In certain research, the MEPs have been considered as indicators of motor cortical excitability in stroke patients (Mäkelä et al., 2015; Jo et al., 2016). Therefore, we utilized MEPs to evaluate the long-term effects of the intervention on motor cortical excitability. The examination procedure in this study adhered to established practice guidelines (Fried et al., 2021). A figure-eight coil (Xiangyu Medical Co., Ltd., Henan, China) was placed over the M1 to elicit the MEPs, while surface electromyography (sEMG) was recorded from the first dorsal interosseous (FDI) muscle. The initial intensity was set at 30% of the maximum stimulator output (MSO). Then the intensity was increased by 5% until the minimum stimulus produced minimal motor-evoked responses (≥50 μV in at least 5 out of 10 trials) in the FDI. The average latency and amplitude of the bilateral MEPs were recorded. The detailed information can be found in Supplementary material S2. If MEPs could not be elicited even at 100% MSO, it was recorded as “NA.” The examination was well tolerated by the patient without any adverse events.

#### 2.4.3 Heart rate variability examination

HRV refers to the fluctuation in the time intervals between adjacent heartbeats. The link between vagus nerve activity and HRV has been established, as the heart is innervated by the vagus motor fibers (Shaffer and Ginsberg, 2017; Capilupi et al., 2020). In our study, we conducted HRV examination to determine whether taVNS effectively targeted the vagus nerve. The average heart rate (HR), the standard deviation of the normal-to-normal (NN) intervals (SDNN), and the square root of the mean squared differences of successive NN intervals (RMSSD) were recorded as time-domain measures of HRV. Additionally, the ratio of low-frequency to high-frequency power (LF/HF ratio) was recorded as a frequency-domain measure of HRV. The detailed processing pipelines for HRV can be found in Supplementary material S2. An increase in parasympathetic activity induced by taVNS correlates with increases in SDNN and RMSSD, and decreases in HR and LF/HF ratio (Marek, 1996). We compared the data collected at T0 with that at T1 and T2 to examine the immediate and long-term effectiveness of taVNS. All the patients completed the examination.

#### 2.4.4 Functional near-infrared spectroscopy (fNIRS) examination

fNIRS is a non-invasive brain imaging technique that detects variations in oxyhemoglobin (HbO2) and deoxyhemoglobin (HbR) within the regional cortex. Increased cortical neural activity leads to escalated metabolic demand, resulting in augmented blood flow in the surrounding vasculature, consequently elevating HbO2 concentrations and reducing HbR concentrations (Huppert et al., 2006; Buxton, 2009). Applying specific near-infrared light, fNIRS can measure brain metabolic alterations associated with neuronal activity (Buxton, 2013; Pinti et al., 2020). Studies have shown strong consistency between fNIRS and functional magnetic resonance imaging (fMRI) (Sasai et al., 2012).

**Figure 2:**
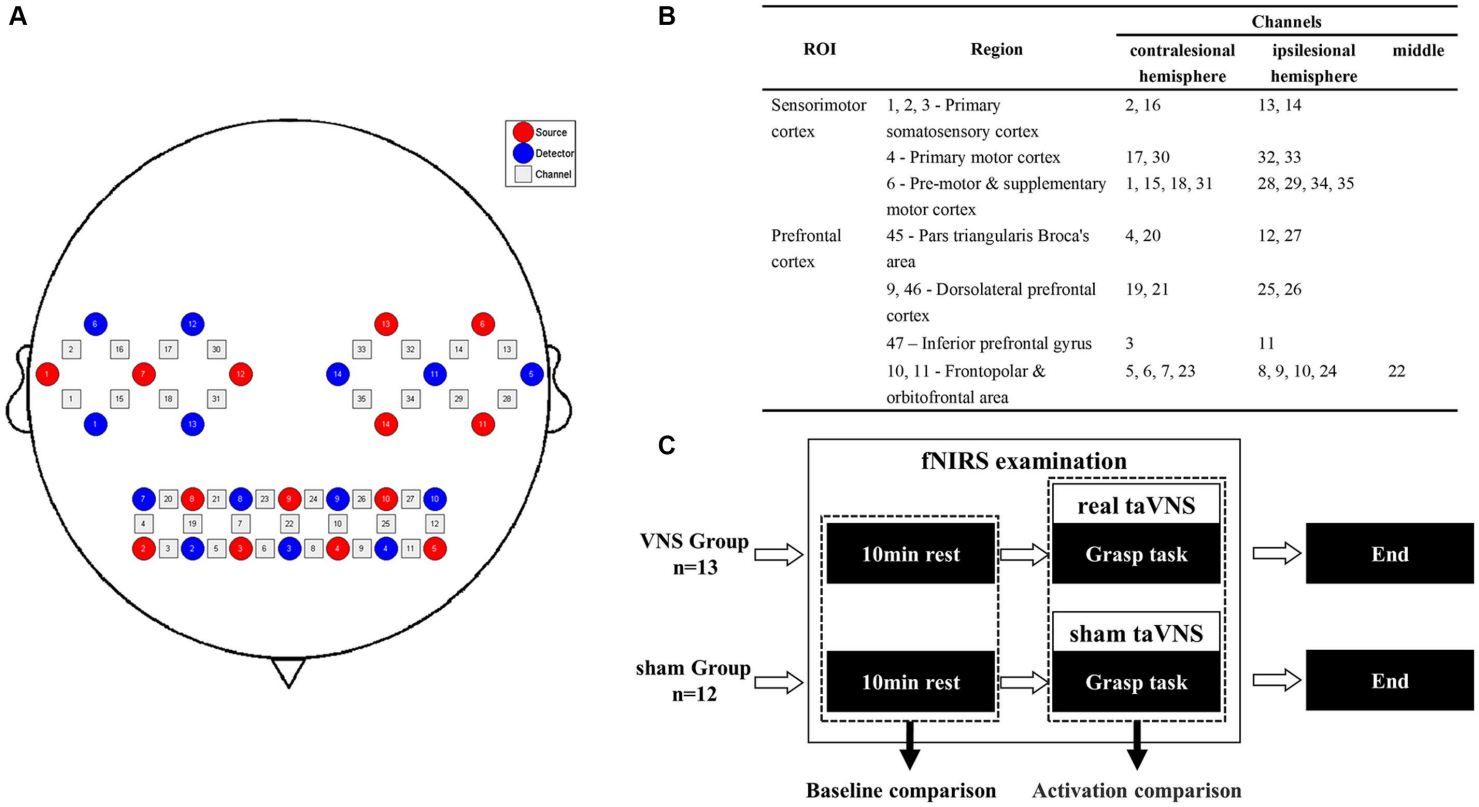
Procedure of the fNIRS examination. **(A)** Channel arrangements with numbers marked on a 2D brain template. **(B)** The regions of interest based on the Brodmann area of each channel. **(C)** Pipeline of the fNIRS examination.

This study used a continuous-wave near-infrared imaging device (NIRSmart II-3000A, Huichuang Medical Co., Ltd., China) consisting of 14 light sources (λ1|2 = 730|850 nm) with an average power of <1 mW and 14 avalanche photodiode detectors operating at 11 Hz sampling rate. The sources and detectors were distributed over the bilateral prefrontal cortex (PFC) and sensorimotor cortex (SMC) according to the 10–20 international standard electrode placement system, constituting 35 channels. The montage of the probes and channels is detailed in Figure 2A. The Patriot localization system was employed to determine the Montreal Neurological Institute (MNI) spatial coordinates for each channel and to annotate the corresponding Brodmann areas. The corresponding brain areas for each fNIRS channel are shown in Figure 2B, Supplementary material S3.

The fNIRS examination was conducted in a quiet room with subdued lighting. To investigate the immediate effect on task-related cortical responses in the patients during the treatment, we simulated a scenario in which patients received taVNS while performing TOT. Initially, a 10-min resting state was recorded, during which patients were instructed to keep their eyes closed, maintain restfulness, and minimize head movements. Subsequently, patients were instructed to perform the grasp task using their hemiplegic hands during the examination, which was practicable and related to the procedure of TOT. The task adopted a block design comprising 20 blocks, each consisting of 15 s of repetitive grasp trials followed by 20 s of rest. Computer-generated auditory cues were provided to guide the patients during the task performance. Patients in the VG received concurrent taVNS, whereas patients in the SG received sham taVNS (Figure 2C).

### 2.5 Data analysis

#### 2.5.1 fNIRS signal processing

The fNIRS signal was processed using MATLAB R2013b (MathWorks, USA), and the Homer2 package was employed for preprocessing the raw data. The following steps were performed: (1) conversion of light intensity to optical density; (2) detection and correction of motion artifacts; (3) application of a bandpass filter (0.01–0.08 Hz) to the signal; (4) conversion of optical density to concentrations of (de)oxygenated hemoglobin based on the modified Beer–Lambert law. Subsequently, the mean concentration of HbO2 during the resting state was calculated to conduct inter-group baseline comparisons. Additionally, the MATLAB-based NIRS-SPM toolkit was utilized to detect brain area activation. To align the data, patients’ hemispheres were flipped, considering the left hemisphere as the ipsilesional hemisphere. Beta values (β) for each channel were calculated using the general linear model (GLM) analysis. Group-level comparison was conducted using an independent t test with a false discovery rate (FDR) correction. Detailed procedures for data processing were shown in Supplementary material S2. BrainNet Viewer (Xia et al., 2013) was employed for 3D visualization of brain activation, and space registration was performed using the NFRI method to convert the channel space into Montreal Neurological Institute (MNI) space.

#### 2.5.2 Clinical data analysis

The statistical analysis was conducted using the open-source statistical software Jamovi (Version 2.4.8) (JAMOVI, 2023). The Shapiro-Wilks test was performed to assess the normality of the data. For baseline demographic and clinical characteristics, the Chi-square test or Fisher’s exact test was used for categorical variables. The two-sample t-test and the Mann–Whitney test were used for continuous variables. A 2 × 2 analysis of covariance (ANCOVA) was used to determine the effects of the treatment on the FMA-UE, ARAT, MBI, and contralesional MEPs parameters, considering time (baseline, post-treatment) as the within-subject factor, group (VNS, Sham) as the between-subjects factor, and baseline data as a covariate. Due to the impairment of cortico-spinal tract integrity after stroke (Yarossi et al., 2019), the statistical analysis for ipsilesional MEP was determined based on the number of MEPs collected. A 3 × 2 repeated measures analysis of variance (RMANOVA) was conducted to determine the effects on the HRV, considering time (T0, T1, T2) as the between-subjects factor and group (VNS, Sham) as the between-subjects factor. A linear regression analysis was used to analyze the correlation between the recovery of upper extremity function and changes in MEPs. A significance level of *p* < 0.05 was considered statistically significant for all tests.

## 3 Results

Forty-three patients were recruited, with 21 allocated to the VG and 21 to the SG. The flow chart can be found in Figure 1. Two participants (one from the VG and another from the SG) dropped out of the study due to unwillingness. In addition, eight participants in the VG and nine participants in the SG were excluded from the fNIRS data analysis. Detailed reasons for participants’ exclusion are shown in Figure 1.

### 3.1 Demographics and clinical characteristics

Table 1 presents a summary of the demographics and clinical characteristics of the patients. There were no statistically significant differences in any of the variables between the two groups. Furthermore, no adverse events were reported throughout the entire duration of the study.

### 3.2 Outcomes for upper extremity function

**Figure 3:**
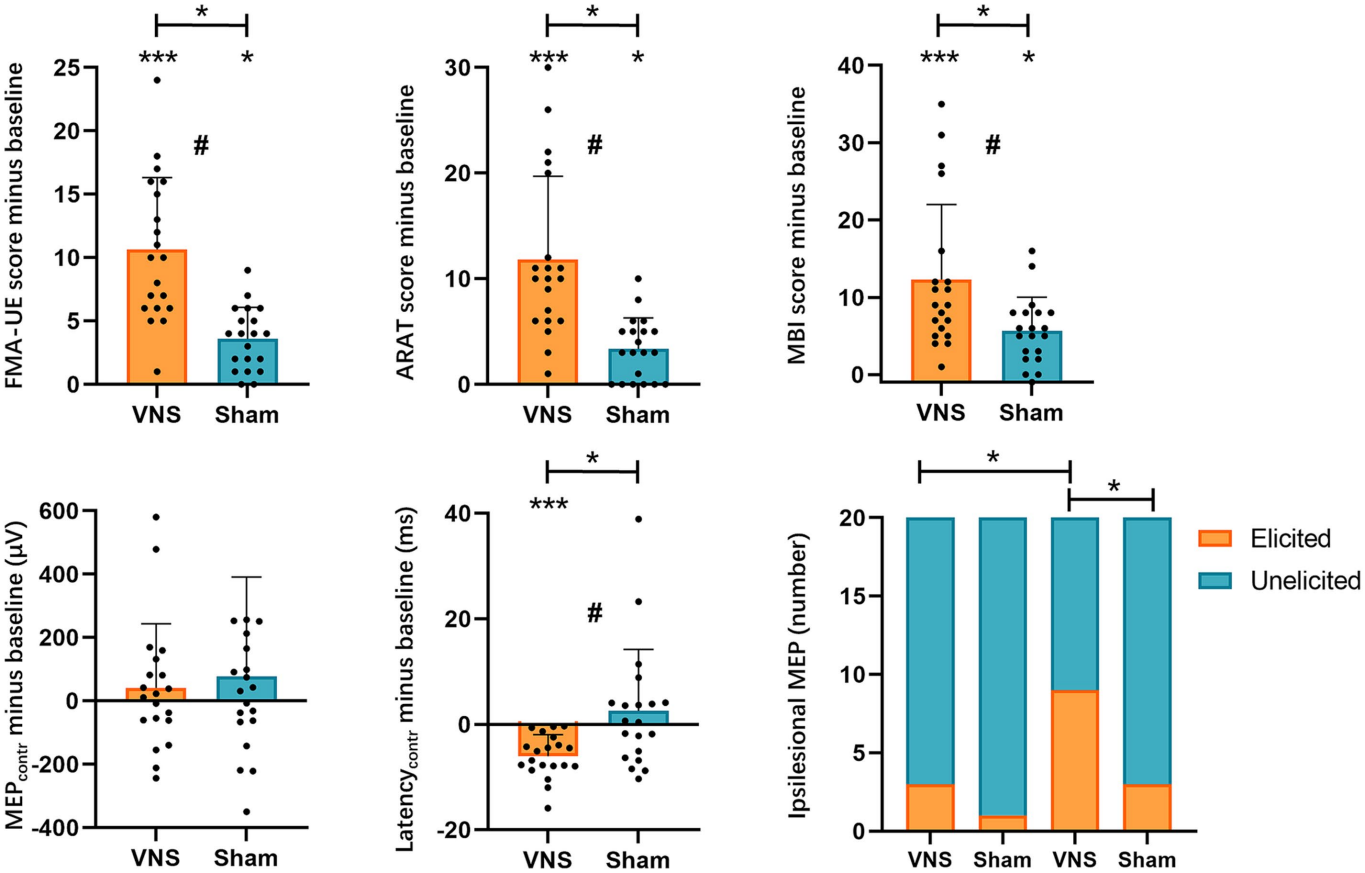
Effects of taVNS on upper extremity function and MEPs. FMA-UE, Fugl-Meyer Assessment-Upper Extremity; ARAT, Action Research Arm Test; MBI, modified Barthel Index; MEPs, motor-evoked potentials; VNS, taVNS group; Sham: Sham group; *, *p* < 0.05; **, *p* < 0.01; ***, *p* < 0.001; #, a significant time × intervention interaction in ANCOVA.

The ANCOVA revealed that patients in both groups showed significant improvements in FMA-UE, ARAT, MBI compared to baseline. Regarding the inter-group comparison, there were statistically greater improvements in FMA-UE, ARAT, and MBI in the VG (Figure 3, Table 2).

**Table 2:** Within-group and between-group comparisons for clinical scales.

Variable	Groups				Within-group differences				Between-group differences	
	Pre-treatment		Post-treatment		Mean (SD)		LS Mean (95% CI)		Difference in LS Mean (95% CI)	*p* value
	VNS	Sham	VNS	Sham	VNS	Sham	VNS	Sham		
FMA-UE^#^	30.5 (8.9)	29.6 (8.7)	41.1 (12)	33.2 (8.3)	10.7 (5.7)	3.6 (2.5)	10.61 (8.63, 12.6)	3.64 (1.65, 5.62)	6.98 (4.26, 9.70)	<0.001
ARAT^#^	15.7 (11)	15.4 (11)	27.5 (15)	18.8 (11)	11.8 (7.9)	3.4 (2.9)	11.84 (9.16, 14.5)	3.36 (0.69, 6.04)	8.47 (4.82, 12.1)	<0.001
MBI^#^	72.2 (17)	69.1 (15)	84.4 (11)	74.8 (14)	12.3 (9.7)	5.7 (4.4)	12.77 (10.2, 15.3)	5.18 (2.61, 7.75)	7.59 (4.07, 11.1)	<0.001

Based on an ANCOVA model after adjusting baseline value. ANCOVA, Analysis of Covariance; CI, Confidence Interval; LS, Least Squares; SD, Standard Deviation. FMA-UE, Fugl-Meyer assessment-upper extremity; ARAT, Action Research Arm Test; MBI, Modified Barthel Index; ^#^significant time × intervention interaction.

### 3.3 Outcomes for MEPs

In our study, only 4 patients were able to elicit ipsilesional MEPs at baseline (VG, *n* = 3; SG, *n* = 1). After 20 sessions of treatment, the number of patients who could elicit ipsilesional MEPs increased to 12 cases (VG, *n* = 9; SG, *n* = 3). To determine the effects on the elicitation rate of ipsilesional MEPs, we employed the Chi-square test or Fisher’s exact test for between-group comparison and the McNemar test for within-group comparison. The Fisher’s exact test indicated that the baseline elicited ipsilesional MEPs were comparable across groups. After treatment, the VG showed significantly higher elicitation rates of ipsilesional MEPs than baseline and SG (Figure 3, Table 3).

**Table 3:** Outcomes for ipsilesional motor-evoked potentials.

Ipsilesional MEPs	Pre-treatment		*p* value	Post-treatment		*p* value
	VNS n (%)	Sham n (%)		VNS n (%)	Sham n (%)	
Elicited	3 (15)	1 (5)	0.605^a^	9 (45)^b*^	3 (15)	0.038^c*^
Unelicited	17 (85)	19 (95)		11 (55)	17 (85)	

MEP, motor evoked potential; ^a^Fisher’s exact test; ^b^McNemar test; ^c^Chi square test; *p < 0.05.

**Figure 4:**
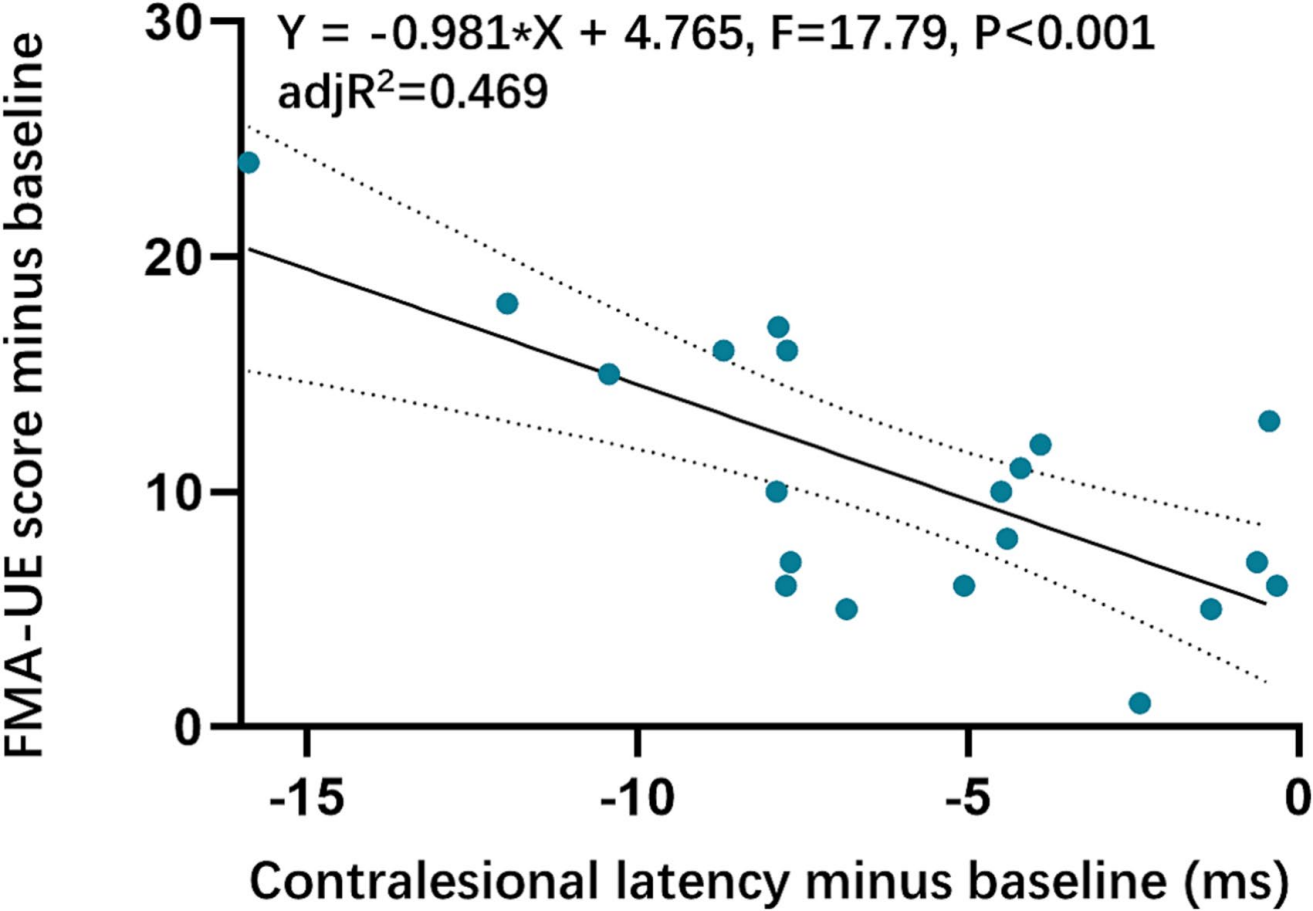
Correlations between Upper extremity function and contralesional MEPs latency. FMA-UE, the Fugl-Meyer Assessment-Upper Extremity.

The contralesional MEPs were collected from all included patients. The ANCOVA found that the contralesional latency of MEPs in the VG significantly shortened compared with baseline. While no significant change in the SG was detected. There was a statistically significant reduction in contralesional latency of MEPs in the VG (Figure 3, Table 4). However, no significant difference was found in contralesional amplitude of MEPs within or between groups (Figure 3, Table 4). Additionally, the univariable regression revealed that there were significant correlations between the improvements in FMA-UE and the changes in the contralesional latency of MEPs (Figure 4).

**Table 4:** Outcomes for contralesional motor-evoked potentials.

Variable	Groups				Within-group differences				Between-group differences	
	Pre-treatment		Post-treatment		Mean (SD)		LS Mean (95% CI)		Difference in LS Mean (95% CI)	*p* value
	VNS	sham	VNS	sham	VNS	sham	VNS	sham		
MEP (μV)	247.5 (143.5)	202.3 (125)	288.8 (180.5)	279.7 (277)	41.4 (202.6)	77.3 (313.9)	62.1 (−45.9, 170.1)	56.6 (−51.4, 164.6)	5.5 (−143.3, 154.3,)	0.943
Latency (ms) ^#^	19.1 (6.7)	17.3 (4.8)	13.1 (6.3)	19.9 (11.3)	−6.0 (4.1)	2.6 (11.7)	−5.7 (−9.6, −1.8)	2.2 (−1.7, 6.1)	−7.9 (−13.3, −2.5)	0.007

Based on an ANCOVA model after adjusting baseline value. ANCOVA, Analysis of Covariance, CI, Confidence Interval, LS, Least Squares, SD, Standard Deviation; MEP, motor-evoked potential amplitude; ^#^significant time × intervention interaction.

### 3.4 Outcomes for fNIRS

**Figure 5:**
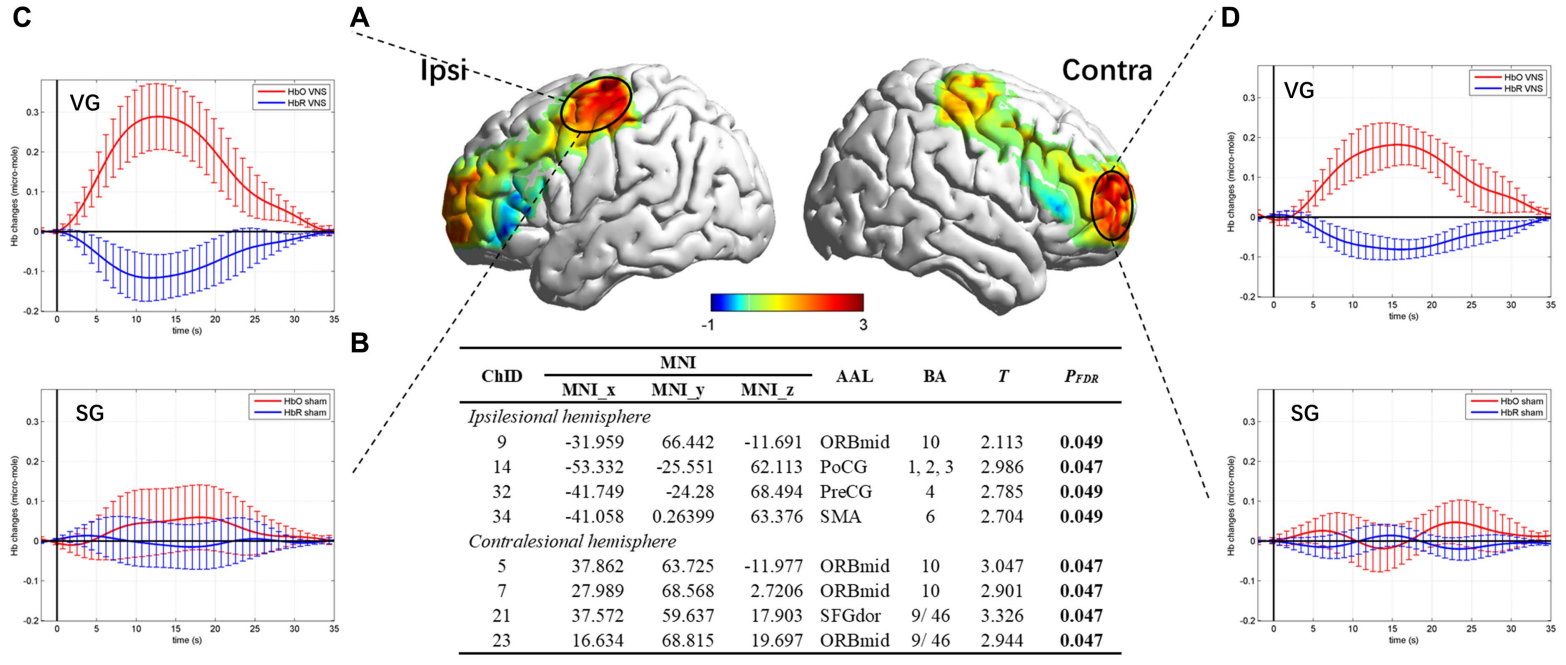
Brain activation map and hemodynamics response for task state. **(A)** Cortical activation map marked on a three-dimensional template (Colin 27 version 2019, using the interpolated mapping algorithm) in ipsilesional and contralesional vision. **(B)** Results from the independent *t*-test of beta values between the VG and the SG. Channels with a significant difference were listed. **(C)** Hemodynamic curve in PoCG_ipsi_. **(D)** Hemodynamic curve in MFG_contr_.

The resting state fNIRS data showed there were no significant differences in baseline. As for task fNIRS data, group-level comparison revealed that the channels in the ipsilesional postcentral gyrus (PoCG_ipsi_, CH14), precentral gyrus (PreCG_ipsi_, CH32), supplementary motor area (SMA_ipsi_, CH34), middle frontal gyrus orbital part (ORBmid_ipsi_, CH9), contralesional orbital middle frontal gyrus (ORBmid_contr_, CH5, CH7, CH23), and dorsolateral superior frontal gyrus (SFGdor_contr_, CH21) showed significantly larger activation in the VG compared to the SG (p_FDR_ < 0.05). The specific coordination of activated channels is shown in Figure 5.

### 3.5 Outcomes for HRV

**Figure 6:**
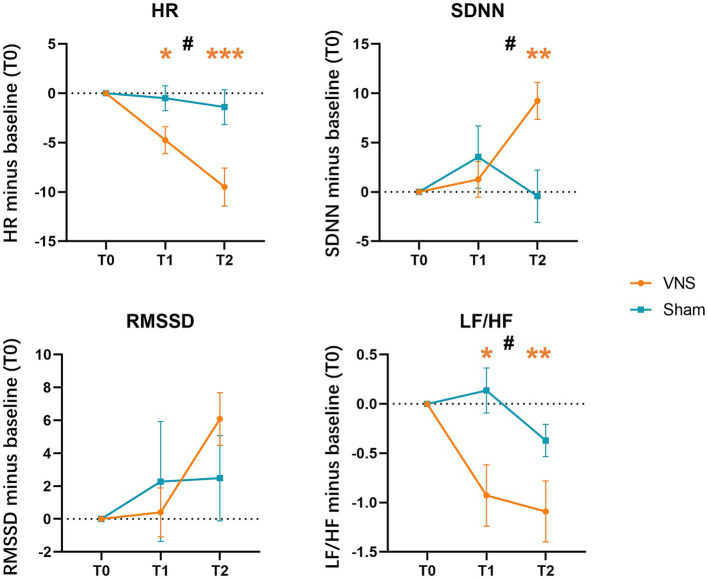
Effects of taVNS on heart rate variability. HR, averaged heart rate; SDNN, the standard deviation of the normal-to-normal intervals; RMSSD, the square root of the mean squared differences of successive normal-to-normal intervals; LF/HF, the ratio of low-frequency to high-frequency power; * in orange color: *p* < 0.05 compared with T0 in VNS group; ** in orange color: *p* < 0.01 compared with T0 in VNS group; *** in orange color: *p* < 0.001 compared with T0 in VNS group; #: a significant time × intervention interaction in repeated-measures ANOVA.

The 3 × 2 RMANOVA revealed significant group*time interactions for HR, SDNN, and LF/HF, but not for RMSSD. We also found significant group main effects in HR and LF/HF. For the interaction effect, Bonferroni *post hoc* tests showed that patients in the VG presented significantly decreased HR at T1, T2 compared with T0, increased SDNN at T2 compared with T0, and decreased LF/HF at T1, T2 compared with T0. However, no between-group difference was found after Bonferroni correction (Figure 6, Table 5).

**Table 5:** Repeated-measures ANOVA for HRV outcomes.

Variable	Group	Descriptive analysis			Within-group differences		Repeated-measures ANOVA			
		T0	T1	T2	T1-T0 in LS Mean (95% CI)	T2-T0 in LS Mean (95% CI)	Effect	F	p	*η*^2^P
HR^a^	VNS	82.5 (11)	77.8 (9.96)	73 (9.56)	−6.47 (−9.8, −3.2)	−10.77 (−15.6, −6.0)	Time	0.866	0.402	0.024
	Sham	78.5 (11.6)	78 (11.4)	77.2 (12.1)	−1.57 (−4.5, 1.4)	−2.16 (−6.5, 2.1)	Time*Group	7.795	0.002	0.178
							Group	10.043	0.003	0.218
SDNN	VNS	29.9 (12.8)	31.2 (11.3)	39.2 (10.8)	4.28 (−1.9, 10.5)	10.84 (5.0, 16.7)	Time	2.190	0.119	0.057
	Sham	28.3 (13)	31.8 (12.7)	27.8 (10.5)	4.90 (−0.7, 10.5)	0.97 (−4.3, 6.2)	Time*Group	6.180	0.003	0.147
							Group	2.370	0.132	0.062
RMSSD	VNS	23.3 (9.92)	23.7 (8.99)	29.3 (9.85)	4.44 (−2.4, 11.3)	7.94 (2.4, 13.5)	Time	0.660	0.520	0.018
	Sham	24.3 (11.7)	26.6 (13.6)	26.8 (7.74)	4.51 (−1.6, 10.6)	3.78 (−1.2, 8.8)	Time*Group	0.865	0.425	0.023
							Group	0.498	0.485	0.014
LF/HF	VNS	2.32 (1.42)	1.39 (0.908)	1.23 (0.849)	−0.60 (−1.3, 0.1)	−0.74 (−1.4, −0.1)	Time	0.328	0.721	0.009
	Sham	1.82 (1.02)	1.96 (1.28)	1.45 (1.02)	0.33 (−0.3, 0.9)	−0.14 (−0.7, 0.4)	Time*Group	3.270	0.044	0.083
							Group	5.732	0.022	0.137

SD, Standard Deviation; LS, Least Squares; CI, Confidence Interval; HR, average heart rate; SDNN, standard deviation of the NN intervals; RMSSD, square root of the mean squared differences of successive NN intervals; LF/HF, low-to-high frequency power ratio; ^a^The Greenhouse–Geisser correction was used for not obeying the sphericity assumption.

## 4 Discussion

In recent years, non-invasive VNS has garnered increasing attention due to its potential to improve upper extremity motor function in stroke patients, although its underlying neural mechanisms are not fully understood. In order to explore the feasibility of the current study, we conducted HRV and fNIRS examinations prior to the initiation of the treatment. The effectiveness of taVNS was assessed using HRV examination. Our current results demonstrated significant decreases in HR, LF/HF ratio, and a marked increase in SDNN in the VG, whereas no changes were observed in the SG. These results indicated that taVNS indeed modulated the vagus nerve network. Subsequently, using fNIRS, we observed greater hemodynamic responses in the bilateral PFC and the ipsilesional SMC during the grasp task with taVNS, suggesting an enhancement of cortical activation. In the fNIRS study conducted by Kunii et al. (2021), the effects of iVNS on cerebral blood flow (CBF) during a resting state and a verbal fluency task were investigated. They found that no changes in CBF were observed during the resting state, while the verbal fluency task led to a significant increase in CBF. Wang et al. (2023) found that a single session of taVNS could significantly bolster the activation within damaged cerebral territories in stroke patients without destabilizing cerebral lateralization. These findings provide evidence that taVNS can effectively activate the task-specific cortex during motor task performance, supporting the immediate effect of taVNS.

The TOT approach aims to teach stroke patients specific task strategies and improve their ability to adapt to the environment through functional tasks related to daily life. Previous fMRI studies (Jang et al., 2003; McCombe Waller et al., 2014) have reported that TOT could improve upper extremity function in post-stroke patients. These studies found that activity in the related motor cortex increased with the recovery of upper extremity motor function. In our study, we observed improvements in the motor capacity of the upper extremity (FMA-UE), arm-hand capacity (ARAT), and activities of daily living (MBI) in both groups. However, patients in the taVNS group demonstrated greater improvements in upper extremity function, which is consistent with previous research (Hoonhorst et al., 2015; Capone et al., 2017; Redgrave et al., 2018; Chang et al., 2021; Li et al., 2022). Notably, the improvement observed in the VG was clinically significant, surpassing the minimal clinically important difference (MCID) of 9–10 points for the FMA-UE in individuals with subacute stroke (Singer and Garcia-Vega, 2017). Khodaparast et al. (2016) observed that the rate of forelimb strength recovery (86%) in ischemic stroke rats was significantly higher after receiving VNS with rehabilitation training compared to the simple rehabilitation group (47%) and the delayed VNS group (42%). Morrison et al. (2019, 2020) reported that intracortical microstimulation with VNS can improve motor cortical plasticity in mice. In this study, significant improvements were observed in upper extremity motor function, indicating that taVNS paired with task-specific activities may promote neuroplastic changes.

Neuroanatomy research has provided insights into the mechanisms underlying the activation of the locus ceruleus-noradrenaline (LC-NE) release system by taVNS. Norepinephrine (NE) is an excitatory neurotransmitter, while the locus coeruleus (LC) is the primary source of the norepinephrine-producing neurons in the brain. Studies indicated that both short-term and long-term VNS can increase neuronal firing rates of the LC, leading to increased NE concentrations in the amygdala, hippocampus, and prefrontal cortex. The VNS-induced NE could maintain long-term activity (Groves et al., 2005; Hulsey et al., 2017). NE can enhance cortical excitability and plasticity, which are associated with daytime vigilance, attention, and motor learning (Duffau, 2006; Ciampa et al., 2022). The fMRI studies by Kraus et al. (2007) and Dietrich et al. (2008) have detected blood oxygenation level-dependent (BOLD) signal activations in the bilateral sensorimotor cortex and prefrontal cortex during taVNS. Furthermore, Frangos and Komisaruk (2017) observed sustained activation of the bilateral precentral gyrus for nearly 10 min (9 min, in fact) following taVNS. Combining these findings with the results of fNIRS in our trial, it is plausible to speculate that taVNS can induce bilateral hemisphere activation during the treatment, which may be attributable to the related neurotransmitter release.

In this study, we utilized single-pulse TMS to assess long-term changes in motor cortex excitability by measuring MEPs in both hemispheres. The study found a relatively low elicitation rate of ipsilesional MEPs in patients at baseline. However, the elicitation rate of ipsilesional MEPs increased significantly from 15% before treatment to 45% after treatment in the VG, which is consistent with previous studies (Escudero et al., 1998; Powell et al., 2019; Yarossi et al., 2019). Moreover, the average latency of ipsilesional MEPs in the VG decreased from 63.53 ms to 55.36 ms, while the amplitude increased from 87.60 μV to 140.18 μV, suggesting a trend toward increased cortical excitability in the ipsilesional M1. Cakar et al. (2016) reported a positive correlation between shorter MEP latency, increased amplitude, and improved motor function in stroke patients. Additionally, we observed a reduction in the latency of contralesional MEPs, indicating the modulation of the contralesional M1. Increased intracortical excitability of contralesional M1 has been observed in subacute and chronic stroke patients (Bütefisch, 2003; Bütefisch et al., 2008), with suggestions that the increased excitability is associated with better recovery of upper extremity function (Bütefisch, 2015). Bi-hemispheric transcranial direct current stimulation (tDCS) studies using fMRI have supported this notion, proposing a cooperative role of contralesional M1 rather than competition (Waters et al., 2017). Furthermore, a randomized controlled trial conducted by Hsu et al. (2023) demonstrated that improvements in FMA-UE scores in subacute stroke patients correlated with changes in functional connectivity within the bilateral intra-hemisphere networks. In our study, we found a significant correlation between the decrease in latency of contralesional MEPs and improvements in FMA-UE scores, which is consistent with previous literature (Wang et al., 2020). Based on these findings, the long-term combined intervention may modulate bilateral motor cortical excitability and facilitate training-specific motor performance.

## 5 Limitations

This study has several limitations that need to be addressed in future research. First, the small sample size and the high dropout rate of the fNIRS examination in this study might result in insufficient statistical power, potentially increasing the chance of a type I error. A larger sample size is needed to confirm these findings and achieve more reliable results. Second, it is worth noting that, due to the limited number of positive events (elicited ipsilesional MEPs), the significance and generalizability of this result should be carefully considered. Finally, follow-up assessments were not conducted to investigate the sustained effects of taVNS in this study, which should be considered in future studies.

## 6 Conclusion

In conclusion, our study provides evidence that the combined intervention of taVNS with TOT is effective in enhancing upper extremity motor function in patients with post-stroke hemiplegia. This improvement can be attributed to the modulation of cortical excitability in both hemispheres, which facilitates the remodeling of motor function.

## Data availability statement

The raw data supporting the conclusions of this article will be made available by the authors, without undue reservation.

## Ethics statement

The studies involving humans were approved by the Ethics Committee of Sir Run Run Hospital, Nanjing Medical University (No. 2020-SR-001). The studies were conducted in accordance with the local legislation and institutional requirements. The participants provided their written informed consent to participate in this study.

## Author contributions

M-HW: Conceptualization, Data curation, Formal analysis, Software, Visualization, Writing – original draft. Y-XW Conceptualization, Writing – review & editing. MX: Investigation, Methodology, Validation, Writing – review & editing. L-YC: Investigation, Methodology, Writing – review & editing. M-FH: Investigation, Methodology, Writing – review & editing. FL: Conceptualization, Project administration, Supervision, Writing – review & editing. Z-LJ: Conceptualization, Funding acquisition, Project administration, Resources, Supervision, Writing – review & editing.
