# Supplementary material for: Transcutaneous auricular vagus nerve stimulation with task-oriented training improves upper extremity function in patients with subacute stroke: a randomized clinical trial
Source: Front Neurosci. 2024 Mar 8;18:1346634. doi: 10.3389/fnins.2024.1346634 (PMC10957639; doi:10.3389/fnins.2024.1346634)
Supplement: Supplementary file 1 [file Data_Sheet_1.DOCX]

Supplementary Material

Transcutaneous auricular vagus nerve stimulation with task-oriented training improves upper extremity function in patients with subacute stroke: a randomized clinical trial

Menghuan Wang^1,2†^, Yixiu Wang^1,2†^, Min Xie^3^, Liyan Chen^1,2^, Mengfei He^1,2^, Feng Lin^2,3^, Zhongli Jiang ^2, 3*^

*** Correspondence:** Zhongli Jiang: [jiangzhongli@njmu.edu.cn](mailto:jiangzhongli@njmu.edu.cn)

#
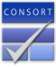
Supplementary 1 (S1)

CONSORT 2010 checklist of information to include when reporting a randomised trial*

| Section/Topic | Item No | Checklist item | Reported on page No |
| --- | --- | --- | --- |
| Title and abstract | | | |
|  | 1a | Identification as a randomised trial in the title | P1 |
|  | 1b | Structured summary of trial design, methods, results, and conclusions (for specific guidance see CONSORT for abstracts) | P1-2 |
| Introduction | | | |
| Background and objectives | 2a | Scientific background and explanation of rationale | P2 |
|  | 2b | Specific objectives or hypotheses | P2 |
| Methods | | | |
| Trial design | 3a | Description of trial design (such as parallel, factorial) including allocation ratio | P3 |
|  | 3b | Important changes to methods after trial commencement (such as eligibility criteria), with reasons | Not need |
| Participants | 4a | Eligibility criteria for participants | P3 |
|  | 4b | Settings and locations where the data were collected | P3 |
| Interventions | 5 | The interventions for each group with sufficient details to allow replication, including how and when they were actually administered | P3-4 |
| Outcomes | 6a | Completely defined pre-specified primary and secondary outcome measures, including how and when they were assessed | P4 |
|  | 6b | Any changes to trial outcomes after the trial commenced, with reasons | None |
| Sample size | 7a | How sample size was determined | Supplementary1 |
|  | 7b | When applicable, explanation of any interim analyses and stopping guidelines | Not need |
| Randomisation: |  |  |  |
| Sequence  generation | 8a | Method used to generate the random allocation sequence | P3 |
|  | 8b | Type of randomisation; details of any restriction (such as blocking and block size) | P3 |
| Allocation  concealment mechanism | 9 | Mechanism used to implement the random allocation sequence (such as sequentially numbered containers), describing any steps taken to conceal the sequence until interventions were assigned | None |
| Implementation | 10 | Who generated the random allocation sequence, who enrolled participants, and who assigned participants to interventions | P3 |
| Blinding | 11a | If done, who was blinded after assignment to interventions (for example, participants, care providers, those assessing outcomes) and how | P3 |
|  | 11b | If relevant, description of the similarity of interventions | P3 |
| Statistical methods | 12a | Statistical methods used to compare groups for primary and secondary outcomes | P4 |
|  | 12b | Methods for additional analyses, such as subgroup analyses and adjusted analyses | Not need |
| Results | | | |
| Participant flow (a diagram is strongly recommended) | 13a | For each group, the numbers of participants who were randomly assigned, received intended treatment, and were analysed for the primary outcome | Figure 1 |
|  | 13b | For each group, losses and exclusions after randomisation, together with reasons | Figure 1 |
| Recruitment | 14a | Dates defining the periods of recruitment and follow-up | P3 |
|  | 14b | Why the trial ended or was stopped | Not need |
| Baseline data | 15 | A table showing baseline demographic and clinical characteristics for each group | Table 1 |
| Numbers analysed | 16 | For each group, number of participants (denominator) included in each analysis and whether the analysis was by original assigned groups | Figure 1 |
| Outcomes and estimation | 17a | For each primary and secondary outcome, results for each group, and the estimated effect size and its precision (such as 95% confidence interval) | Table 2-5 |
|  | 17b | For binary outcomes, presentation of both absolute and relative effect sizes is recommended | Not need |
| Ancillary analyses | 18 | Results of any other analyses performed, including subgroup analyses and adjusted analyses, distinguishing pre-specified from exploratory | Not need |
| Harms | 19 | All important harms or unintended effects in each group (for specific guidance see CONSORT for harms) | None |
| Discussion | | | |
| Limitations | 20 | Trial limitations, addressing sources of potential bias, imprecision, and, if relevant, multiplicity of analyses | P9 |
| Generalisability | 21 | Generalisability (external validity, applicability) of the trial findings | P9 |
| Interpretation | 22 | Interpretation consistent with results, balancing benefits and harms, and considering other relevant evidence | P8-9 |
| Other information | | |  |
| Registration | 23 | Registration number and name of trial registry | None |
| Protocol | 24 | Where the full trial protocol can be accessed, if available | None |
| Funding | 25 | Sources of funding and other support (such as supply of drugs), role of funders | P10 |

Citation: Schulz KF, Altman DG, Moher D, for the CONSORT Group. CONSORT 2010 Statement: updated guidelines for reporting parallel group randomised trials. BMC Medicine. 2010;8:18.

© 2010 Schulz et al. This is an Open Access article distributed under the terms of the Creative Commons Attribution License (<http://creativecommons.org/licenses/by/2.0>), which permits unrestricted use, distribution, and reproduction in any medium, provided the original work is properly cited.

*We strongly recommend reading this statement in conjunction with the CONSORT 2010 Explanation and Elaboration for important clarifications on all the items. If relevant, we also recommend reading CONSORT extensions for cluster randomised trials, non-inferiority and equivalence trials, non-pharmacological treatments, herbal interventions, and pragmatic trials. Additional extensions are forthcoming: for those and for up-to-date references relevant to this checklist, see [www.consort-statement.org](http://www.consort-statement.org).

**Sample size:**

To determine the sample size for our study, we calculated it using analysis of covariance (ANCOVA) in Gpower3.1 software. The effect size was calculated with reference to the research results of Wu et al. (1), using FMA-UE as the measurement index. Assuming a minimum power of 0.95, type I error level at 0.05, and considering 20% drop out rate, it was determined that each group needed at least 15 subjects, resulting in a total of 30 subjects. A total of 40 subjects were included in this study, meeting the minimum sample size requirement (2).

1. Wu D, Ma J, Zhang L, Wang S, Tan B, Jia G. Effect and Safety of Transcutaneous Auricular Vagus Nerve Stimulation on Recovery of Upper Limb Motor Function in Subacute Ischemic Stroke Patients: A Randomized Pilot Study. *Neural Plasticity* (2020) 2020:1–9. doi: 10.1155/2020/8841752

2. Lenhard W, Lenhard A. Computation of Effect Sizes. Psychometrica (2022). doi: 10.13140/RG.2.2.17823.9232

# Supplementary 2 (S2)

## The processing pipeline for HRV data (1) ：

The electrocardiography recordings were conducted for 5 minutes with patients in a seated position. The data were subsequently analyzed by SA-3000P^Ⓡ^ (Medicore Inc., Seoul, Korea). The detailed methods are as follows:

(1) Average heart rate (HR): The R waves were identified from the electrocardiography signal, which represent the depolarization of the ventricles, with exclusion of any abnormal artifacts. Then the R-R intervals, the time between successive R waves, were calculated. The average heart rate was determined as the mean of multiple R-R intervals, as indicated by the following formula:

*HR=*
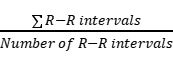


(2) Standard deviation of the normal-to normal interval (SDNN): The R-R intervals were extracted from successive normal heartbeats. The N-N intervals, obtained by measuring R-R intervals, were then used to calculate the standard deviation of the N-N intervals. The formula is as follows.

*SDNN=*
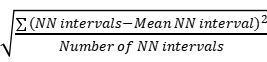


(3) Root mean square of successive differences (RMSSD): The differences between consecutive N-N intervals were calculated, followed by the determination of the average of the squared value for each successive difference. Then took the square root of the mean of these squared differences. The formula is as follows.

*RMSSD=*
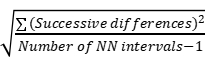


(4) Low-frequency to high-frequency ratio (LF/HF): Firstly, the time-domain data was transformed into frequency-domain components using Fourier analysis. Then the power with low-frequency band (0.04~0.15 Hz) as well as high-frequency band (0.15~0.40 Hz) were identified. Finally, the LF/HF ratio was computed by taking the ratio of the power in the low-frequency band to the power in the high-frequency band. The formula is as follows.

*LF/HF=*
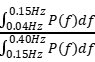
 *, where P(f) is the power spectral density function.*

## The processing pipeline for MEPs data (2,3) ：

For motor-evoked potential (MEP) calculation, surface electromyography (sEMG) was applied to record electrical potentials from the first dorsal interosseous (FDI) muscle. The detailed parameters were as follows: a sampling rate of 5000 Hz, an amplifier set at 500x, a notch filter at 50 Hz, and a low-pass filter at 500 Hz. Five waveforms with good repeatability and representativeness were selected for analysis. MEP latency refers to the time taken for the motor response to occur after the stimulation of the motor cortex. It is calculated by measuring the time interval between the onset of stimulation and the onset of the MEP waveform. MEP amplitude refers to the magnitude or size of the electrical potential recorded from the muscles following stimulation. It is calculated by measuring the peak-to-peak amplitude, which is the difference between the peak and the trough of the MEP waveform.


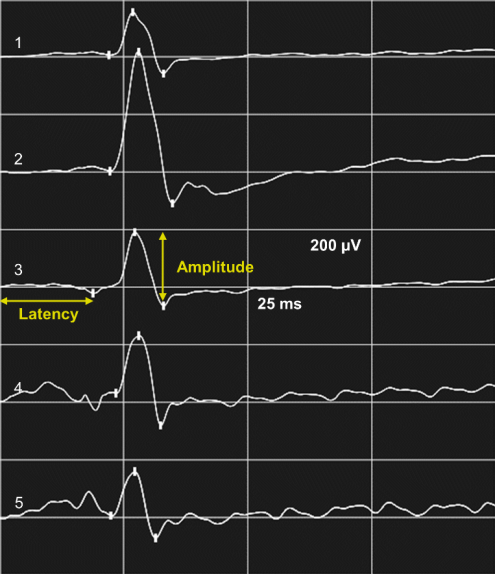


## The processing pipeline for fNIRS data

### Data preprocessing

Signal processing for functional near-infrared spectroscopy (fNIRS) was conducted in the MATLAB R2013b environment (MathWorks, USA), utilizing the open-source toolkit Homer2 (version 2.8) for signal preprocessing. The preprocessing procedures were as follows:

(1) Channels with poor signal quality were removed through visual inspection.

(2) Converted the intensity (raw data) to optical density.

(3) A sliding window methodology was applied to traverse the signal for the channel-by-channeled identification of motion artifacts. The time window was set at 0.5 seconds, with signal changes exceeding 20 times the standard deviation or 5 times the amplitude within the window being marked as motion artifacts.

(4) Motion artifacts were corrected through a kurtosis-based wavelet algorithm within a ± 1 second window around the identified artifacts. The kurtosis level was set at 3.3 (4).

(5) A bandpass filter was applied to eliminate low frequency drift and high frequency physiological noise. The frequency band was set from 0.01 to 0.08 Hz, which is considered highly relevant to brain neural activity (5).

(6) The processed of optical density changes was further converted to concentration changes of oxygenated hemoglobin (HbO2) and deoxygenated hemoglobin (HbR) through the modified Beer-Lambert law (6). Herein, the differential pathlength factor (DPF) was set at 6 cm for both wavelengths, and the distance between the light source and the probe (r) was 30 mm.

(7) A correlation-based signal improvement of the concentration changes was performed to further adjustment of the blood flow signals. Given that HbO2 is considered the most sensitive indicator reflecting changes in cortical activity, only HbO2 was extracted for subsequent analysis (7).

### GLM analysis: task-based brain activation analysis

To ensure consistent analysis, the data was converted to mirror the patients’ flipped hemispheres. The left hemisphere was considered the ipsilateral hemisphere. A first-level analysis using the general linear model (GLM) was conducted with the NIRS_SPM toolbox. The GLM design matrix defined onset as the beginning of each block with a duration of 20 seconds. The hemodynamic response function with time and dispersion derivatives was selected as the basis function to model the hemodynamic response. Detrending was performed using the wavelet minimum description length (wavelet-MDL) algorithm (8). Precoloring method using the hrf was applied to attenuate high frequency components (9). The GLM estimated parameters (β values), considered the primary signal magnitude indicator, were calculated for each channel (10).

For group-level analysis, the β values of each channel were averaged over the patients in each group. Subsequently, an independent t-test was conducted for between-group comparison with false discovery rate (FDR) correction. The significance threshold was set at PFDR<0.05.

## Reference

1. Heart rate variability. Standards of measurement, physiological interpretation, and clinical use. Task Force of the European Society of Cardiology and the North American Society of Pacing and Electrophysiology. *Eur Heart J* (1996) 17:354–381.

2. Rossi S, Antal A, Bestmann S, Bikson M, Brewer C, Brockmöller J, Carpenter LL, Cincotta M, Chen R, Daskalakis JD, et al. Safety and recommendations for TMS use in healthy subjects and patient populations, with updates on training, ethical and regulatory issues: Expert Guidelines. *Clinical Neurophysiology* (2021) 132:269–306. doi: 10.1016/j.clinph.2020.10.003

3. Vucic S, Stanley Chen K-H, Kiernan MC, Hallett M, Benninger DH, Di Lazzaro V, Rossini PM, Benussi A, Berardelli A, Currà A, et al. Clinical diagnostic utility of transcranial magnetic stimulation in neurological disorders. Updated report of an IFCN committee. Clin Neurophysiol (2023) 150:131–175. doi: 10.1016/j.clinph.2023.03.010

4. Am C, El M, M F, G G. A kurtosis-based wavelet algorithm for motion artifact correction of fNIRS data. *NeuroImage* (2015) 112: doi: 10.1016/j.neuroimage.2015.02.057

5. Biswal B, Zerrin Yetkin F, Haughton VM, Hyde JS. Functional connectivity in the motor cortex of resting human brain using echo-planar mri. *Magn Reson Med* (1995) 34:537–541. doi: 10.1002/mrm.1910340409

6. Delpy DT, Cope M, van der Zee P, Arridge S, Wray S, Wyatt J. Estimation of optical pathlength through tissue from direct time of flight measurement. *Phys Med Biol* (1988) 33:1433–1442. doi: 10.1088/0031-9155/33/12/008

7. Cui X, Bray S, Reiss AL. Functional near infrared spectroscopy (NIRS) signal improvement based on negative correlation between oxygenated and deoxygenated hemoglobin dynamics. *Neuroimage* (2010) 49:3039–3046. doi: 10.1016/j.neuroimage.2009.11.050

8. Jang KE, Tak S, Jung J, Jang J, Jeong Y, Ye JC. Wavelet minimum description length detrending for near-infrared spectroscopy. *J Biomed Opt* (2009) 14:034004. doi: 10.1117/1.3127204

9. Worsley KJ, Friston KJ. Analysis of fMRI time-series revisited--again. *Neuroimage* (1995) 2:173–181. doi: 10.1006/nimg.1995.1023

10. Kim DH, Lee K-D, Bulea TC, Park H-S. Increasing motor cortex activation during grasping via novel robotic mirror hand therapy: a pilot fNIRS study. *J NeuroEngineering Rehabil* (2022) 19:8. doi: 10.1186/s12984-022-00988-7

# Supplementary 3 (S3)

| **The MNI coordinates and anatomical labels corresponding to the channels** | | | | | | |
| --- | --- | --- | --- | --- | --- | --- |
| **Channel number (S-D)** | **MNI** | | | **Brodmann area overlap** | **Proportion** | **ROI** |
|  | **x** | **y** | **z** |  |  |  |
| CH1 (S1-D1) | 60 | -5 | 46 | 6 - Pre-Motor and Supplementary Motor Cortex | 0.52632 | SMC |
|  |  |  |  | 4 - Primary Motor Cortex | 0.29699 |  |
| CH2 (S1-D6) | 62 | -30 | 52 | 1 - Primary Somatosensory Cortex | 0.46241 | SMC |
|  |  |  |  | 3 - Primary Somatosensory Cortex | 0.033835 |  |
|  |  |  |  | 4 - Primary Motor Cortex | 0.045113 |  |
| CH3 (S2-D2) | 53 | 44 | -12 | 45 - pars triangularis Broca's area | 0.6744 | PFC |
|  |  |  |  | 46 - Dorsolateral prefrontal cortex | 0.3256 |  |
|  |  |  |  | 47 - Inferior prefrontal gyrus | 0.55755 |  |
| CH4 (S2-D7) | 59 | 31 | 4 | 45 - pars triangularis Broca's area | 0.83067 | PFC |
| CH5 (S3-D2) | 38 | 64 | -12 | 11 - Orbitofrontal area | 0.40234 | PFC |
|  |  |  |  | 47 - Inferior prefrontal gyrus | 0.34375 |  |
|  |  |  |  | 10 - Frontopolar area | 0.17188 |  |
| CH6 (S3-D3) | 15 | 71 | -12 | 11 - Orbitofrontal area | 0.96321 | PFC |
| CH7 (S3-D8) | 28 | 69 | 3 | 11 - Orbitofrontal area | 0.50492 | PFC |
|  |  |  |  | 10 - Frontopolar area | 0.49508 |  |
| CH8 (S4-D3) | -9 | 72 | -11 | 11 - Orbitofrontal area | 0.87031 | PFC |
|  |  |  |  | 10 - Frontopolar area | 0.12969 |  |
| CH9 (S4-D4) | -32 | 66 | -12 | 11 - Orbitofrontal area | 0.63498 | PFC |
|  |  |  |  | 47 - Inferior prefrontal gyrus | 0.18251 |  |
|  |  |  |  | 10 - Frontopolar area | 0.13308 |  |
| CH10 (S4-D9) | -20 | 72 | 4 | 10 - Frontopolar area | 0.74086 | PFC |
|  |  |  |  | 11 - Orbitofrontal area | 0.25914 |  |
| CH11 (S5-D4) | -52 | 44 | -14 | 47 - Inferior prefrontal gyrus | 0.65809 | PFC |
|  |  |  |  | 46 - Dorsolateral prefrontal cortex | 0.27206 |  |
| CH12 (S5-D10) | -59 | 27 | 1 | 44 - pars opercularis, part of Broca's area | 0.0095847 | PFC |
|  |  |  |  | 45 - pars triangularis Broca's area | 0.61342 |  |
| CH13 (S6-D5) | -62 | -27 | 51 | 3 - Primary Somatosensory Cortex | 0.27839 | SMC |
|  |  |  |  | 1 - Primary Somatosensory Cortex | 0.24542 |  |
|  |  |  |  | 2 - Primary Somatosensory Cortex | 0.1978 |  |
| CH14 (S6-D11) | -53 | -26 | 62 | 3 - Primary Somatosensory Cortex | 0.592 | SMC |
|  |  |  |  | 1 - Primary Somatosensory Cortex | 0.356 |  |
|  |  |  |  | 2 - Primary Somatosensory Cortex | 0.008 |  |
| CH15 (S7-D1) | 51 | -5 | 56 | 6 - Pre-Motor and Supplementary Motor Cortex | 0.72689 | SMC |
|  |  |  |  | 4 - Primary Motor Cortex | 0.27311 |  |
| CH16 (S7-D6) | 52 | -27 | 63 | 3 - Primary Somatosensory Cortex | 0.48627 | SMC |
|  |  |  |  | 1 - Primary Somatosensory Cortex | 0.35686 |  |
|  |  |  |  | 2 - Primary Somatosensory Cortex | 0.058824 |  |
| CH17 (S7-D12) | 41 | -27 | 69 | 4 - Primary Motor Cortex | 0.6426 | SMC |
|  |  |  |  | 6 - Pre-Motor and Supplementary Motor Cortex | 0.057762 |  |
| CH18 (S7-D13) | 41 | -4 | 64 | 6 - Pre-Motor and Supplementary Motor Cortex | 0.86765 | SMC |
|  |  |  |  | 4 - Primary Motor Cortex | 0.13235 |  |
| CH19 (S8-D2) | 48 | 55 | 1 | 46 - Dorsolateral prefrontal cortex | 0.79845 | PFC |
|  |  |  |  | 10 - Frontopolar area | 0.20155 |  |
| CH20 (S8-D7) | 52 | 41 | 19 | 45 - pars triangularis Broca's area | 0.88235 | PFC |
|  |  |  |  | 46 - Dorsolateral prefrontal cortex | 0.11765 |  |
| CH21 (S8-D8) | 38 | 60 | 18 | 46 - Dorsolateral prefrontal cortex | 0.57724 | PFC |
|  |  |  |  | 10 - Frontopolar area | 0.42276 |  |
| CH22 (S9-D3) | 5 | 72 | 3 | 10 - Frontopolar area | 0.99367 | PFC |
| CH23 (S9-D8) | 17 | 69 | 20 | 10 - Frontopolar area | 1 | PFC |
| CH24 (S9-D9) | -11 | 70 | 20 | 10 - Frontopolar area | 1 | PFC |
| CH25 (S10-D4) | -47 | 56 | 3 | 46 - Dorsolateral prefrontal cortex | 0.80769 | PFC |
|  |  |  |  | 10 - Frontopolar area | 0.19231 |  |
| CH26 (S10-D9) | -34 | 60 | 20 | 46 - Dorsolateral prefrontal cortex | 0.68127 | PFC |
|  |  |  |  | 10 - Frontopolar area | 0.31873 |  |
| CH27 (S10-D10) | -54 | 39 | 19 | 45 - pars triangularis Broca's area | 0.99281 | PFC |
| CH28 (S11-D5) | -61 | -3 | 44 | 6 - Pre-Motor and Supplementary Motor Cortex | 0.50182 | SMC |
|  |  |  |  | 4 - Primary Motor Cortex | 0.29091 |  |
| CH29 (S11-D11) | -51 | -1 | 56 | 6 - Pre-Motor and Supplementary Motor Cortex | 0.968 | SMC |
| CH30 (S12-D12) | 31 | -25 | 74 | 4 - Primary Motor Cortex | 0.66775 | SMC |
|  |  |  |  | 6 - Pre-Motor and Supplementary Motor Cortex | 0.31922 |  |
| CH31 (S12-D13) | 30 | -4 | 70 | 6 - Pre-Motor and Supplementary Motor Cortex | 1 | SMC |
| CH32 (S13-D11) | -42 | -24 | 68 | 4 - Primary Motor Cortex | 0.65942 | SMC |
| CH33 (S13-D14) | -32 | -22 | -22 | 4 - Primary Motor Cortex | 0.52843 | SMC |
|  |  |  |  | 6 - Pre-Motor and Supplementary Motor Cortex | 0.47157 |  |
| CH34 (S14-D11) | -41 | 0 | 63 | 6 - Pre-Motor and Supplementary Motor Cortex | 0.98496 | SMC |
| CH35 (S14-D14) | -30 | 1 | 68 | 6 - Pre-Motor and Supplementary Motor Cortex | 0.97902 | SMC |

ROI: region of interests; PFC: prefrontal cortex; SMC: sensorimotor cortex.
